# CCR2^−^ and CCR2^+^ corneal macrophages exhibit distinct characteristics and balance inflammatory responses after epithelial abrasion

**DOI:** 10.1038/mi.2016.139

**Published:** 2017-01-25

**Authors:** J Liu, Y Xue, D Dong, C Xiao, C Lin, H Wang, F Song, T Fu, Z Wang, J Chen, H Pan, Y Li, D Cai, Z Li

**Affiliations:** 1 Integrated Chinese and Western Medicine Postdoctoral Research Station, Jinan University, Guangzhou, China; 2 International Ocular Surface Research Center and Institute of Ophthalmology, Jinan University Medical School, Guangzhou, China; 3 Key Laboratory for Regenerative Medicine, Ministry of Education, Jinan University, Guangzhou, China; 4 Department of Medical Images, The Third People’s Hospital, Puyang, China; 5 Section of Leukocyte Biology, Department of Pediatrics, Children’s Nutrition Research Center, Baylor College of Medicine, Houston, Texas, USA

## Abstract

Macrophages are distributed throughout the body and are crucial for the restoration of damaged tissues. However, their characteristics in the cornea and roles in the repair of corneal injures are unclear. Here we show that corneal macrophages can be classified as CCR2^−^ macrophages, which already exist in the cornea at embryonic day 12.5 (E12.5) and are similar to yolk sac-derived macrophages, microglia, in phenotype and gene expression, and CCR2^+^ macrophages, which do not appear in the cornea until E17.5. At a steady state, CCR2^−^ corneal macrophages have local proliferation capacity and are rarely affected by monocytes; however, following corneal epithelial abrasion, most CCR2^−^ corneal macrophages are replaced by monocytes. In contrast, CCR2^+^ macrophages are repopulated by monocytes under both a steady-state condition and following corneal wounding. Depletion of CCR2^+^ macrophages decreases corneal inflammation after epithelial abrasion, whereas depletion of CCR2^−^ macrophages increases inflammation of the injured cornea. Loss of either cell type results in a delay in corneal healing. These data indicate that there are two unique macrophage populations present in the cornea, both of which participate in corneal wound healing by balancing the inflammatory response.

## Introduction

Clear vision depends on an accurate refractive device. The cornea, located at the anterior segment of the eye, is responsible for light refraction and transmission. Corneal integrity is maintained by a variety of cells, and their imbalance can result in serious eye diseases. Therefore, research on cells involved in maintaining corneal integrity is of the utmost importance. Recent research has highlighted the roles of immune cells in the repair of injured tissues.^1, 2, 3, 4, 5, 6, 7, 8^ Of these immune cells, macrophages appear to be particularly concerned.

Located throughout the body, macrophages are evolutionarily conserved phagocytes. Historically, tissue-resident macrophages have been considered to originate from circulating, adult monocytes.^9, 10^ However, this theory has become controversial in recent years as considerable evidence has revealed that some macrophages settle in tissues during the early embryonic stage. These cells have local proliferation capacity, and their long-term maintenance rarely depends on renewal from blood monocytes.^11, 12^ More recent studies have confirmed that tissue-resident macrophages can be categorized into three populations based on their origins. The first macrophage population derives from the early erythromyeloid progenitors (EMPs), which are located in the yolk sac. At embryonic days 7.5–11.5 (E7.5–11.5), these progenitors migrate into peripheral tissues and directly differentiate into macrophages without going through an intermediate monocyte state. The second macrophage population derives from the late EMPs that migrate into the fetal liver and express the transcription factor c-Myb. At E11.5–16.5, these progenitors become monocytes and migrate into peripheral tissues where they further differentiate into macrophages. The third macrophage population derives from hematopoietic stem cells (HSCs), which are located in the developing fetal liver or bone marrow. At E17.5 or after birth, HSCs differentiate into monocytes and migrate into peripheral tissues where a proportion of these monocytes differentiate into macrophages.^13, 14^ Thus, macrophages are a heterogeneous cell population, and each population of macrophages migrates into peripheral tissues during different stages of development. Although previous studies confirmed that macrophages are distributed in the cornea,^15, 16^ the specific biological characteristics of corneal macrophages such as heterogeneity, origin and renewal mechanisms are still unclear.

The role of macrophages in promoting healing of injured tissues has been widely studied. For example, when the liver is damaged, macrophages phagocytize cell fragments and activate the related signaling pathways to promote the differentiation of liver progenitor cells for repair of the injured tissues. Deficiency of liver macrophages delays the liver repair process.^17, 18^ In addition, during the process of repairing injured skin, macrophages promote angiogenesis and wound closure. Depletion of these cells results in a decrease of vascularized granulation tissue formation and serious wound bleeding, ultimately hindering the healing process.^19^ Moreover, after cardiac ischemic injury, the loss of macrophages causes myocardial rupture and delays the repair process.^20^ However, due to the heterogeneity of macrophages, each population tends to have different roles in repairing damaged tissues, and this complicates research on the function of macrophages.^20, 21^ According to their active states and gene expression profiles, macrophages can be classified into M1 type (the classical activation state) and M2 type (the alternative activation sate). M1 macrophages can secrete inflammatory factors and have a proinflammatory role. M2 macrophages are anti-inflammatory and promote tissue repair and remodeling.^21, 22, 23, 24^ To this point, research on macrophages in corneal wound healing has been limited and thus, the roles of each population of macrophages in corneal wound healing have been unclear.

To this end, we analyzed the heterogeneity of corneal macrophages, explored their origin by comparing phenotypes and gene expression between corneal macrophages and microglia, and compared the maintenance mechanisms of corneal macrophages at a steady state with those occurring during wound healing using a bone marrow transplantation model. Finally, we investigated the respective roles of each macrophage population in corneal wound healing by depleting each population with antibodies or an antagonist.

## Results

### The adult cornea contains distinct macrophage populations

**Figure 1:**
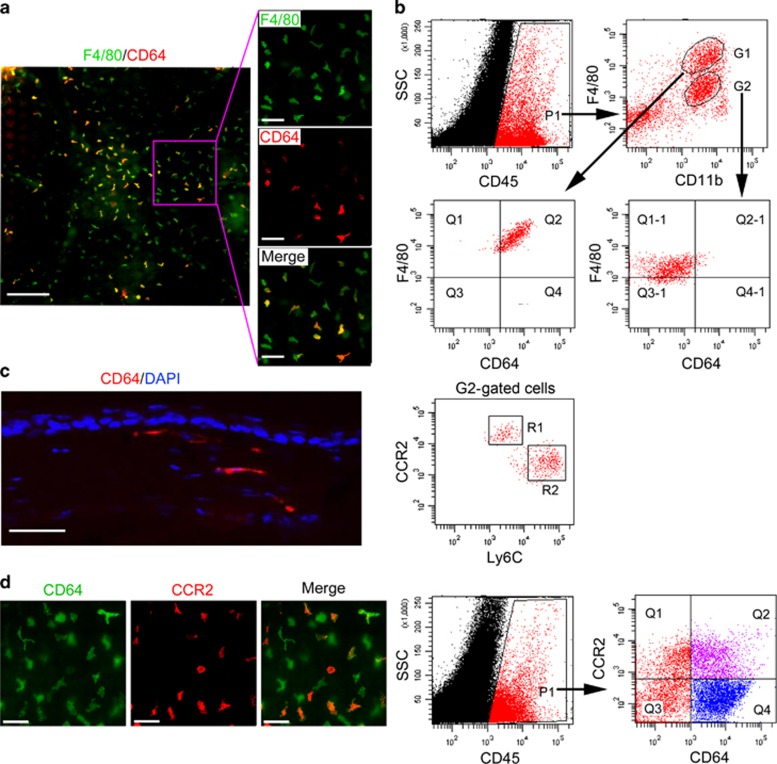
Identification of corneal macrophages. (**a**) Corneal whole-mount immunostaining of anti-mouse F4/80-FITC and CD64-PE (bars=left image, 200 μm; right three images, 100 μm). (**b**) Flow cytometric analysis of corneal macrophages. CD45^+^ cells (P1 gated) within corneal cells from 8-week-old mice were analyzed for F4/80 and CD11b antigens, and F4/80^+^CD11b^+^ cells were classified into F4/80^high^ CD11b^+^ (G1 gated) and F4/80^low^ CD11b^+^ (G2 gated) subsets. G1- and G2-gated cells were then analyzed for CD64 antigen expression as shown on the x axis. G2-gated cells were further analyzed for the expression of CCR2 and Ly6C antigens. (**c**) Anti-mouse CD64-PE and DAPI costaining of corneal section (bar=50 μm). (**d**) CD64-FITC and CCR2-APC costaining of cornea (left three images, bars=100 μm). CD45^+^ corneal cells (P1 gated) from 8-week-old mice were analyzed for the expression of CD64 and CCR2 antigens (right two images). The gating strategy in this figure was justified by related isotype controls, as detailed in the Supplementary Information online. All samples in this figure were obtained at the same time from 8-week-old mice. APC, allophycocyanin; DAPI, 4′, 6-diamidino-2-phenylindole; FITC, fluorescein isothiocyanate; PE, phycoerythrin; SSC, side scatter.

F4/80 and CD11b antigens have previously been used to detect macrophages and monocytes in some tissues.^15, 16, 25, 26^ In this study, corneal macrophage identity was confirmed using the highly specific macrophage marker, CD64.^27^ Using whole-mount immunostaining of the cornea, we found that a large number of F4/80^+^ cells were distributed throughout the cornea, and some of the F4/80^+^ cells were also CD64^+^ (Figure 1a). Using flow cytometric analysis to investigate CD45, F4/80 and CD11b expression of the corneal cells, F4/80^+^ CD11b^+^ cells in the cornea were classified into G1 (F4/80^high^ CD11b^+^) and G2 (F4/80^low^ CD11b^+^) subsets, and only G1 subset cells were CD64^+^. G2 subset cells (CD64^−^) expressed CCR2 and Ly6C, the monocyte-related antigens, and were classified into CCR2^high^ Ly6C^low^ (R1) and CCR2^low^ Ly6C^high^ (R2) two subsets (Figure 1b). Therefore, of the F4/80^+^ CD11b^+^ cells in the cornea, only F4/80^high^ CD11b^+^ cells are macrophages and the F4/80^low^ CD11b^+^ cells are monocytes. In addition, immunostaining of corneal sections revealed that CD64^+^ macrophages are located in the corneal stromal layers (Figure 1c).

Owing to differences in origin and the tissue microenvironment, tissue-resident macrophages are highly heterogeneous. For example, cardiac macrophages can be distinguished into those of adult monocyte origin or embryonic origin by their CCR2 expression and dependence.^28^ Similarly, costaining with anti-CD64 and -CCR2 antibodies revealed that corneal macrophages can also be classified into CCR2^−^ and CCR2^+^ groups (Figure 1d).

### CCR2^−^ and CCR2^+^ macrophages migrate into the cornea at different embryonic stages

**Figure 2:**
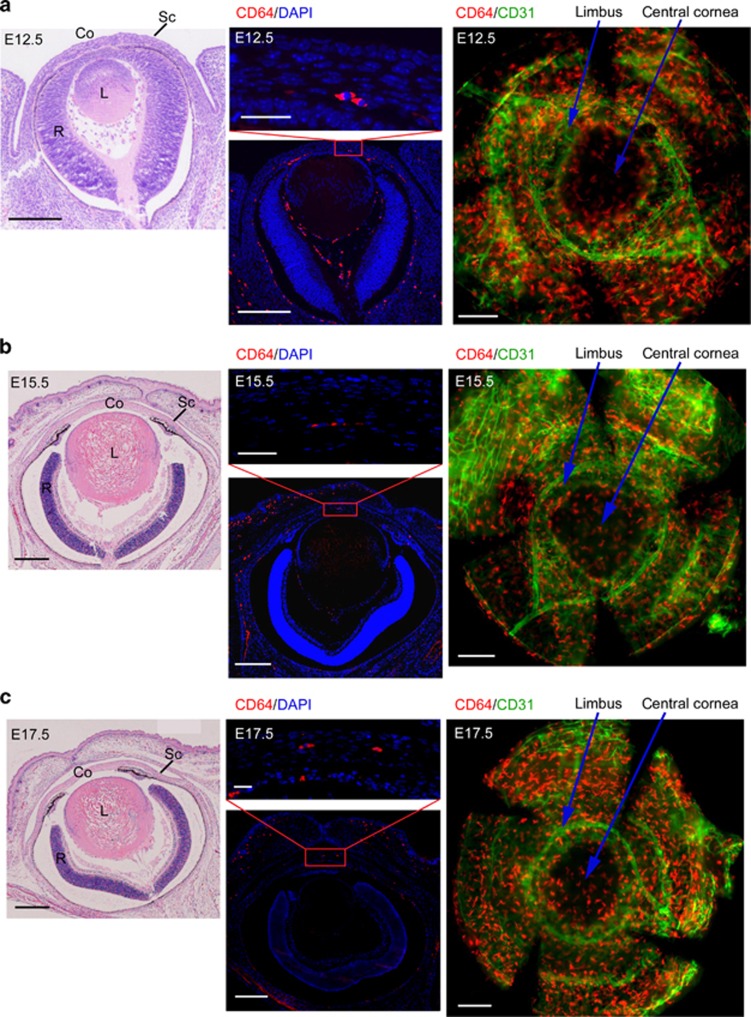

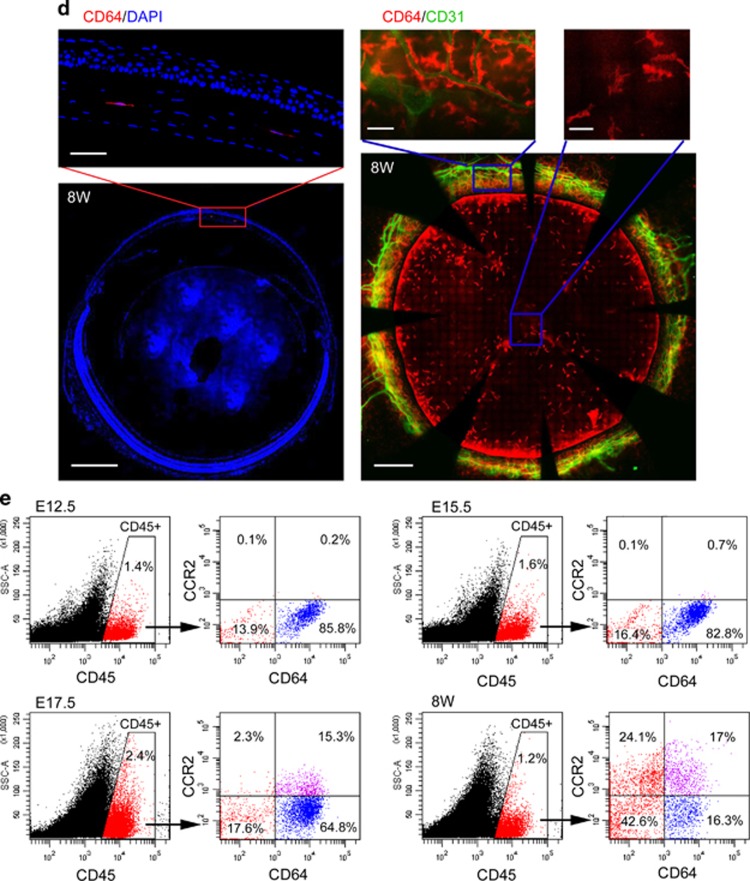
Presence of macrophages during corneal development. (**a**) E12.5, (**b**) E15.5, and (**c**) E17.5 H&E staining of eye section (bars=left images, 200 μm). Anti-mouse CD64-PE antibody and DAPI costaining of eye sections (bars=upper-middle images, 25 μm; lower-middle images, 200 μm). Whole-mount immunostaining of cornea with anti-mouse CD64-PE and CD31-FITC antibodies (bars=right images, 200 μm). (**d**) Anti-mouse CD64-PE antibody and DAPI costaining of eye section from 8-week-old mice (bars=left upper image, 50 μm; left lower image, 500 μm). Whole-mount immunostaining of corneas from 8-week-old mice with anti-mouse CD64-PE and CD31-FITC antibodies (bar=right upper two images, 50 μm; right lower image, 500 μm). (**e**) Flow cytometric analysis of corneal macrophages from embryonic and 8-week-old mice. All samples in this figure were obtained at the same time. Co, cornea; DAPI, 4′, 6-diamidino-2-phenylindole; E, embryonic day; FITC, fluorescein isothiocyanate; H&E, hematoxylin and eosin; L, lens, R, retina; Sc, sclera; SSC, side scatter.

Some macrophages can settle in peripheral tissues during the early embryonic stage and persist in adult tissues.^12, 28, 29, 30, 31, 32, 33^ At E12.5, the lens detaches from the ectoderm, leaving a space. Subsequently, mesenchymal cells begin to migrate into the space, finally forming the corneal stromal layer and endothelium.^34^ We have confirmed that CD64^+^ macrophages settled in the corneal stromal layers (Figure 1c). It is still unclear whether the macrophages that migrate into the cornea are accompanied by mesenchymal cells at this time. Hematoxylin and eosin staining of eyeball sections from E12.5 mice revealed that the eyelids are not closed at this point of development and a presumptive cornea exists (Figure 2a). Immunostaining of these sections and whole-mount cornea showed that CD64^+^ macrophages already existed in the cornea at this early point in development, and they were located among posterior mesenchymal cells (Figure 2a). Moreover, immunostaining of eye sections and whole-mount cornea from E15.5 and E17.5 revealed that CD64^+^ macrophages already migrated into the stromal layer, and many macrophages were distributed in the central cornea with some cells located around the limbal blood vessels (Figure 2b,c). Immunostaining of eye sections and corneal whole mounts from 8-week-old mice revealed that CD64^+^ macrophages were still presented in the central cornea and corneal limbus of adult mouse cornea (Figure 2d). In addition, flow cytometric analysis of corneal cells from embryonic and adult mice revealed that only CCR2^−^ macrophages are present in the corneas of E12.5 and E15.5 mice, and CCR2^+^ macrophages do not appear in the cornea until E17.5 (Figure 2e).

### CCR2^−^ corneal macrophages are similar to microglia in phenotype and gene expression

**Figure 3:**
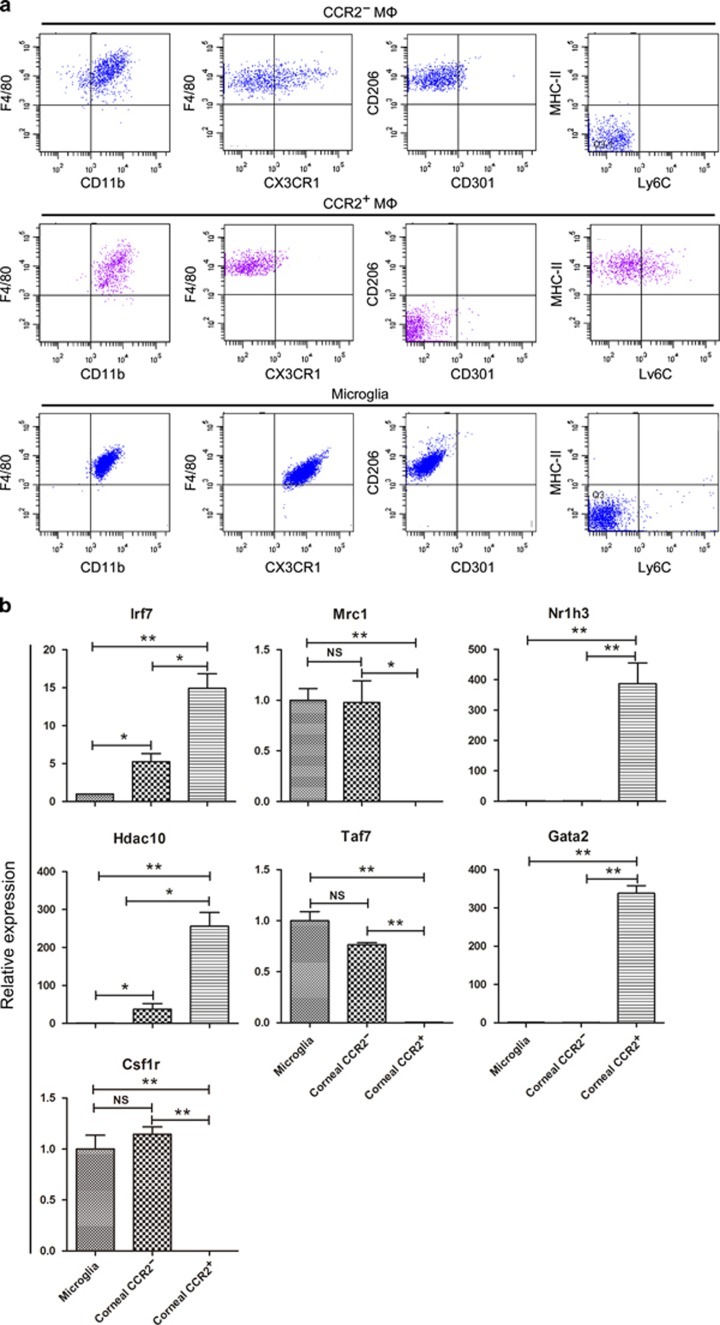
Comparison of corneal macrophages and microglia. (**a**) Analysis of surface antigen expression in CCR2^−^ corneal macrophages, CCR2^+^ corneal macrophages, and microglia. The gating strategy was set using the related isotype controls as detailed in the Supplementary Information online. (**b**) Analysis of *Irf7*, *Nr1h3*, *Hdac10*, *Taf7*, *Gata2*, and *Csf1r* gene expression in corneal macrophages and microglia. Expression levels are shown relative to the measurement in the microglia, which served as a control (*n*=three independent experiments, 10 mice per experiment). The results are presented as mean±s.d. *P* values were calculated using unpaired Student’s *t*-test: *P<0.05 and **P<0.01. All samples in this figure were obtained at the same time from 8-week-old mice. MHC, major histocompatibility complex; NS, nonsignificant.

CCR2^−^ macrophages already exist in the cornea at E12.5, a time at which definitive hematopoiesis occurs in fetal liver, but not bone marrow.^35^ Therefore, CCR2^−^ macrophages found in the presumptive cornea may derive from progenitors originating in the fetal liver or earlier yolk sac. Previous studies have revealed that microglia in the brain are exclusively derived from yolk sac progenitors.^11, 28, 29, 30, 36^ To explore the origin of corneal macrophages, we analyzed the expression of macrophage common antigens^28^ and genes^27^ between corneal macrophages and microglia. The results indicated that the phenotype of CCR2^−^ macrophages (F4/80^+^ CD11b^+^ CX3CR1^+/−^ CD206^+^ CD301^−^ MHC-II^−^ Ly6C^−^) was similar to that of microglia (F4/80^+^ CD11b^+^ CX3CR1^+^ CD206^+^ CD301^−^ MHC-II^−^ Ly6C^−^), whereas CCR2^+^ macrophages (F4/80^+^ CD11b^+^ CX3CR1^−^ CD206^−^ CD301^−^ MHC-II^+^ Ly6C^+/−^) was not (Figure 3a). In addition, the expression pattern of *Mrc1*, *Nr1h3*, *Taf7*, *Gata2*, and *Csf1r* was similar between CCR2^−^ macrophages and microglia, whereas the expression of these genes in CCR2^+^ macrophages differed significantly from microglia. Although in CCR2^−^ macrophages and microglia, *Irf7* and *Hdac10* gene expression were different, both of these genes were expressed at low levels in CCR2^−^ macrophages and microglia compared with CCR2^+^ macrophages (Figure 3b).

### CCR2^−^ and CCR2^+^ corneal macrophages have distinct maintenance mechanisms

**Figure 4:**
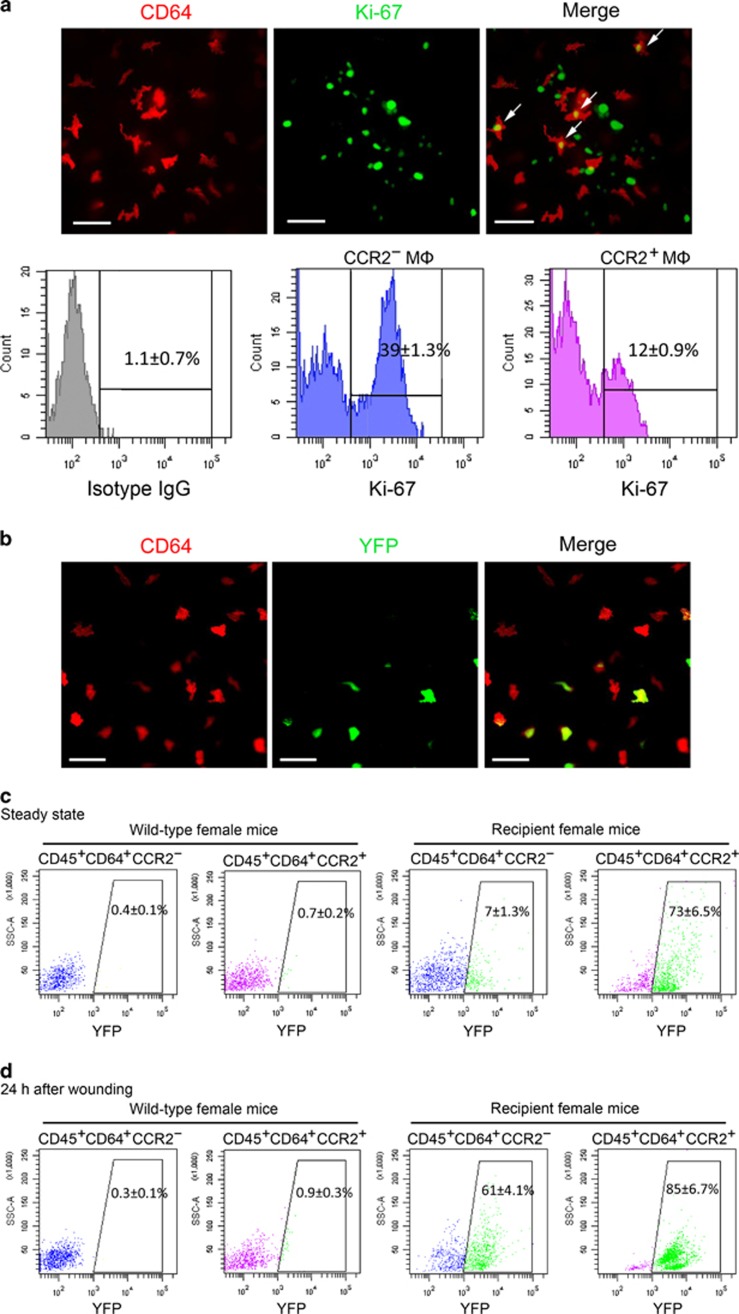
Maintenance mechanisms of corneal macrophages at steady state or after corneal wounding. (**a**) Costaining of cornea from 8-week-old mice with CD64-PE and Ki-67-FITC (the upper three images) (bars=50 μm). Flow cytometric analysis of the percentage of Ki-67-positive cells in CCR2^−^ and CCR2^+^ corneal macrophages (lower three images). Control isotype antibodies are shown on the left. (**b**) Staining of cornea from recipient female mice with anti-mouse CD64-PE (bars=100 μm). (**c**) Percentage of YFP^+^ cells in corneal macrophages from wild and recipient mice at steady state, and (**d**) after corneal epithelial wounding. All samples in this figure were obtained at the same time. FITC, fluorescein isothiocyanate; IgG, immunoglobulin G; PE, phycoerythrin; SSC, side scatter; YFP, yellow fluorescent protein.

Traditionally, the renewal of macrophages is considered to depend on the continuous contribution of blood monocytes.^37, 38^ However, many studies have shown that some tissue-resident macrophages have local proliferation capacity, and their long-term maintenance does not rely on the contribution of blood monocytes.^35, 36^ In our study, whole-mount immunostaining of the cornea revealed that some corneal macrophages were positive for the cell proliferation marker Ki-67. Further flow cytometric analysis showed that while the percentage of Ki67^+^ cells in the CCR2^−^ corneal macrophages was 39±1.3%, it was 12±0.9% in the CCR2^+^ macrophages (Figure 4a). To determine whether corneal macrophages depend on the contribution of blood monocytes for their long-term maintenance, we transplanted the bone marrow of Rosa-YFP male mice into irradiated female mice. At 1 month after bone marrow transplantation, the *Sry* gene, located on the Y chromosome, was found to be expressed in peripheral blood leukocytes from recipient female mice (Supplementary Figure 1a online), indicating successful bone marrow transplantation. Furthermore, wild-type female mice did not have YFP^+^ cells in their peripheral blood leukocytes, whereas the percentage of YFP^+^ cells in the peripheral blood leukocytes of the recipient female mice reached 93% (Supplementary Figure 1b). At 3 months after transplantation, whole-mount immunostaining of corneas from the recipient mice revealed that some CD64^+^ macrophages were YFP positive, indicating that these macrophages were derived from blood monocytes of the donor. However, there were still some corneal macrophages that did not express YFP, indicating that these macrophages were derived from the host (Figure 4b). Further flow cytometric analysis showed that the percentage of YFP^+^ cells in the CCR2^−^ macrophage population was only 7±1.3%, whereas in the CCR2^+^ macrophages they were 73±6.5% (Figure 4c). These results suggest that at steady state, renewal of corneal CCR2^−^ macrophages rarely depends on blood monocyte repopulating, whereas the renewal of corneal CCR2^+^ macrophages is highly dependent on the infusion of blood monocytes.

Various tissue-resident macrophages are known to use distinct maintenance mechanisms under different conditions. For example, cardiac macrophages are able to self-renew through local proliferation at steady state, but when they are depleted or under a state of cardiac inflammation, monocytes contribute to all macrophage populations.^28^ Therefore, we sought to determine whether the maintenance mechanism used by corneal macrophages changed after corneal wounding. We found that 24 h after wounding, the percentage of YFP^+^ cells in CCR2^−^ macrophages had risen to 61±4.1%, and the percentage of YFP^+^ cells in CCR2^+^ macrophages had risen to 85±6.7% (Figure 4d). This indicates that the maintenance mechanism of CCR2^−^ macrophages changes after corneal wounding.

### Depletion of CCR2^−^ and CCR2^+^ corneal macrophages delays corneal wound healing

**Figure 5:**
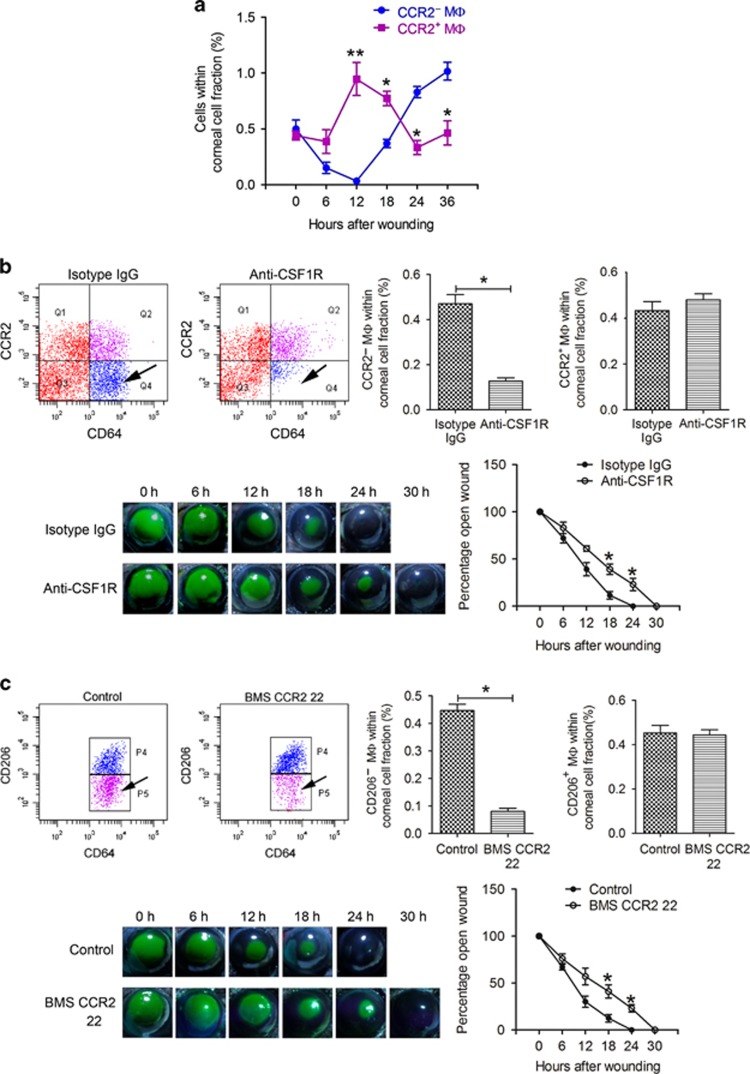
The effect of macrophage depletion on corneal epithelial wound healing. (**a**) Dynamic changes in corneal macrophages after corneal epithelial wounding (*n*=three independent experiments, 10 mice per experiment). *P* values were calculated using unpaired Student’s *t*-test: *P<0.05 and **P<0.01. (**b**) Percentage of CCR2^−^ and CCR2^+^ macrophages in cornea cells measured after 10 subconjunctival injections of isotype immunoglobulin G or anti-CSF1R antibody (upper two images and bar diagram) (*n*=4 independent experiments, 10 mice per experiment). Corneal epithelial wounds were stained with sodium fluorescein (lower left images), and the stained area was measured using Photoshop CS4 (Adobe company, San Jose, CA). The graph depicts the dynamic change in the area of open wounds after corneal epithelial wounding. The percentage of the wound open was obtained by dividing the original wound area by the area of the wound at each time point (*n*=12 mice at each time point). Unpaired Student’s *t*-test was used to analyse differences between groups at each time point after wounding, *P<0.05. Factorial-design ANOVA was performed to analyze differences between two groups at the whole level by antibody injection and time point after wounding, *P*=0.03. (**c**) The percentage of CD206^−^ macrophages (CCR2^+^ macrophages) and CD206^+^ macrophages (CCR2^−^ macrophages) in cornea cells was measured after 10 intraperitoneal injections of the diluent for BMS CCR2 22 or BMS CCR2 22 (upper two images and bar diagram) (*n*=4 independent experiments, 10 mice per experiment). Corneal epithelial wounds were stained with sodium fluorescein at the indicated time points (lower left images), and the percentage of open wound at each time point was calculated as shown (*n*=12 mice at each time point). Unpaired Student’s *t*-test was used to analyze differences between groups at each time point after wounding, *P<0.05. Factorial-design ANOVA was performed to analyze differences between two groups at the whole level by antagonist injection and time point after wounding, *P*=0.03.The time that samples were obtained in the experiment groups and control groups were the same for each time point. ANOVA, analysis of variance.

Macrophages, which are important innate immune cells, may promote the healing of injured tissues through phagocytizing cell debris, participating in the inflammatory response, and releasing specific cytokines and growth factors.^19^ After corneal epithelial abrasion, we found opposing dynamic changes in two subsets of macrophages: the percentage of CCR2^−^ macrophages in corneal cells initially decreased and were undetectable 12 h after wounding, and then reappeared and gradually increased, reaching a higher percentage than CCR2^+^ macrophages at 24 and 36 h after wounding. In contrast, the percentage of CCR2^+^ macrophages increased after wounding, reaching a peak at 12 h, and then began to gradually decrease, reaching the original percentage 36 h after wounding (Figure 5a).

The signaling pathway downstream of the colony-stimulating factor receptor (CSF1R) is responsible for the development and proliferation of macrophages. In CSF1R^−/−^ mice, yolk sac-derived macrophages are seriously defective.^28, 30^ In our study, 10 continuous, subconjunctival injections of anti-CSF1R antibody decreased the number of CCR2^−^ corneal macrophages significantly, whereas CCR2^+^ macrophages were not affected (Figure 5b). After corneal epithelial abrasion of these mice, the rate of wound closure was markedly lower than in the isotype immunoglobulin G control group (Figure 5b). In addition, we confirmed that the long-term maintenance of CCR2^+^ cells is highly dependent on the contribution of blood monocytes (Figure 4c,d). Defects in the CCR2 receptor hinder the migration of monocytes.^39^ To observe the effect of CCR2 deficiency on CCR2^+^ corneal macrophages, we injected the CCR2 antagonist, BMS CCR2 22, intraperitoneally. Because the CCR2 antagonist also blocks the binding of the anti-CCR2 antibody, CCR2^+^ macrophages could not be identified with anti-CCR2 antibody in these studies. We have confirmed that CCR2^−^ macrophages express CD206, whereas CCR2^+^ macrophages do not (Figure 3a), and thus used the anti-CD206 antibody to differentiate CCR2^−^ and CCR2^+^ macrophages. Ten continuous injections of BMS CCR2 22 decreased the number of CD206^−^ (CCR2^+^) macrophages significantly, whereas the CD206^+^ (CCR2^−^) macrophages were not affected (Figure 5c). Moreover, after corneal epithelial abrasion, the rate of wound closure was obviously lower in these mice than in the control group (Figure 5c).

### Macrophage subsets participate in corneal wound healing by balancing the inflammatory response

**Figure 6:**
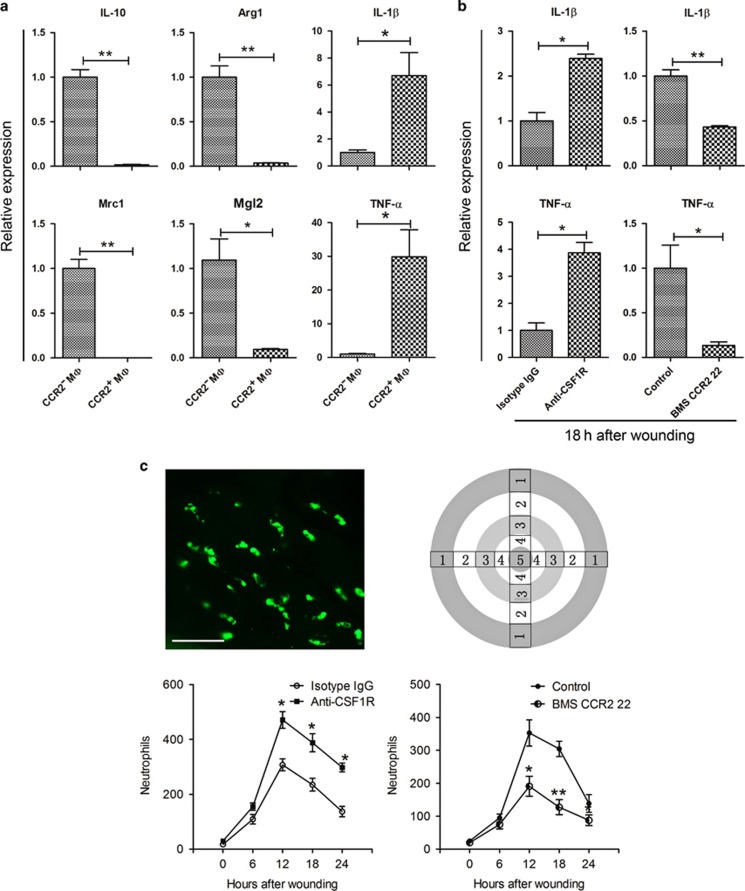
Changes in corneal inflammation following CCR2^+^ or CCR2^-^ macrophage depletion. (**a**) Analysis of M1/M2 gene expression profiles in flow cytometry-sorted CCR2^−^ and CCR2^+^ corneal macrophages (*n*=three independent experiments, 10 mice per experiment). Expression levels are shown relative to the measurement for CCR2^−^ macrophages, which served as a control. (**b**) Changes in inflammatory cytokine (*IL-1β and TNF-α*) expression in cornea after CCR2^−^ or CCR2^+^ corneal macrophage depletion (*n*=three independent experiments, 6 mice per experiment). (**c**) Changes in neutrophil recruitment to cornea after CCR2^−^ or CCR2^+^ corneal macrophage depletion. To observe neutrophil recruitment, corneas with complete limbi were stained with anti-Ly6G-FITC (upper-left image, bar=50 μm). Neutrophils were counted in zones 1–5 (upper right image) in four quadrants of each cornea, and the total cell number of these counts was plotted against the time after wounding (*n*=6 mice at each time point). The results are presented as mean±s.d. *P* values were calculated using unpaired Student’s *t*-test: *P<0.05 and **P<0.01. The time that samples were obtained in the experiment groups and control groups were the same for each time point. IgG, immunoglobulin G; IL, interleukin; TNF-α, tumor necrosis factor-α.

Macrophages are a heterogeneous cell population with unique roles in pathological conditions.^28, 40^ According to their activation state, they can be divided into M1 macrophages (the classical activation state), which participate in the proinflammatory response, and M2 macrophages (the alternative activation state), which have roles in inhibiting the inflammatory response.^21, 22, 23, 24^ Each macrophage activation state has a different gene expression profile. The canonical genes expressed by M1 macrophages are *IL-1β* and *TNF-α*, the genes typically expressed by M2 macrophages are *IL-10*, *Arg1*, *Mrc1*, *Mgl1*, *Mgl2*, *Ym1*, and *Fizz1*.^41, 42^ Quantitative PCR of flow cytometry-sorted corneal macrophages showed that CCR2^−^ macrophages express *IL-10*, *Arg1*, *Mrc1*, and *Mgl2*, and CCR2^+^ cells express *IL-1β* and *TNF-α* (Figure 6a). Therefore, considering the opposite dynamic changes in the two macrophage populations during the corneal healing process, we speculated that CCR2^+^ corneal macrophages may promote early inflammation after corneal wounding, and CCR2^−^ corneal macrophages may inhibit inflammation at a later time to support the corneal healing process. To address this, we further investigated changes in corneal inflammation following depletion of corneal macrophages and found that at 18 h after corneal epithelial abrasion, injection of anti-CSF1R antibody significantly increased inflammation of the cornea, expression of inflammatory cytokines *IL-1β* and *TNF-α*, and the presence of neutrophils in the cornea to levels higher than in the isotype immunoglobulin G control group. Moreover, injection of the CCR2 antagonist, BMS CCR2 22, significantly decreased inflammation of the cornea, expression of inflammatory cytokines *IL-1β* and *TNF-α* and the presence of neutrophils in the cornea to levels lower compared with that of the control group (Figure 6b,c).

## Discussion

Macrophages, located throughout the body, have long been known to be crucial for the repair of damaged tissue. Nevertheless, macrophages in the cornea have not been well characterized. In this study, we identified two macrophage populations. The CCR2^−^ population is present in the cornea at E12.5, whereas the CCR2^+^ population does not appear in the cornea until E17.5. These populations not only display different phenotypes, gene expression profiles, and maintenance mechanisms, but also participate in corneal wound healing in different ways. CCR2^+^ macrophages demonstrate a proinflammatory ability at the early stage of corneal wound healing, whereas CCR2^−^ macrophages exert anti-inflammatory effects during the later stage. Moreover, deficiency of either population results in a delay in corneal wound healing.

Using whole-mount immunostaining of the cornea and flow cytometric analysis, we identified CD64^+^ macrophages in the cornea, and categorized these cells into CCR2^−^ and CCR2^+^ populations. Recent studies have revealed that tissue-resident macrophages arise from three origins: c-Myb^−^ EMPs, located in the yolk sac; c-Myb^+^ EMPs, located in the fetal liver; and HSCs, located in the fetal liver or bone marrow. While both c-Myb^+^ EMPs and HSCs differentiate into monocytes before becoming macrophages, c-Myb^−^ EMPs directly differentiate into macrophages without passing through the monocyte stage.^11, 12, 14, 35, 43, 44, 45^ Many studies have demonstrated that microglia exclusively originate from the c-Myb^−^ EMPs in the yolk sac.^11, 13, 28, 29, 30, 36^ Comparison of corneal CCR2^−^ and CCR2^+^ macrophages and microglia in terms of phenotype and gene expression showed that only CCR2^−^ corneal macrophages are similar to microglia. Therefore, CCR2^−^ corneal macrophages may arise from the same progenitor as microglia, whereas CCR2^+^ corneal macrophages may originate from c-Myb^+^ EMPs or HSCs. Further study will be required to determine if this is the case.

Although many studies have confirmed that tissue-resident macrophages can be maintained not only by blood monocyte infusion but also by self-renewal, the underlying mechanistic details remain unclear. Macrophages in lung tissue can re-establish themselves through local proliferation, which rarely relies on monocyte contributions, regardless of whether they are at steady state or have been conditionally depleted.^46^ While cardiac macrophages are chiefly maintained through local proliferation, following macrophage depletion or during cardiac inflammation, Ly6C^hi^ monocytes contribute to all cardiac macrophage populations.^28^ Moreover, macrophages residing in the intestine are continually replaced by blood monocytes, even at steady state.^47^ In our study, we found that CCR2^−^ corneal macrophages were primarily maintained through local proliferation, and were rarely replaced by donor blood monocytes. In contrast, CCR2^+^ corneal macrophages had lower proliferation ability and were largely replaced by donor monocytes. However, after corneal epithelial abrasion, a large proportion of the CCR2^−^ corneal macrophages were replaced by donor monocytes. Therefore, CCR2^−^ corneal macrophages are similar to cardiac macrophages in that their mechanism of maintenance can be altered under different conditions. Moreover, CCR2^+^ corneal macrophages are similar to intestinal macrophages in that they are maintained through continuous contributions of blood monocytes, even at steady state. Thus, distinct maintenance mechanisms of corneal macrophages exist depending on the tissue microenvironment and physiological setting.

Healing of damaged tissues is a complicated process involving inflammation, cell migration and proliferation, protein synthesis, wound closure, and tissue remodeling.^48, 49, 50^ Macrophages are heterogeneous and different subsets have distinct responses in the healing process. Davies *et al.*^26, 51^ observed that in mild zymosan peritonitis of mice, a large number of inflammatory monocyte-derived macrophages could be found in the inflamed peritoneal tissue during the period of acute neutrophil influx, whereas few F4/80^high^-resident macrophages were seen. However, at later time points, F4/80^high^-resident macrophages reappeared in this tissue.^26, 51, 52^ This macrophage response is characterized as a *disappearance reaction*, and can be the result of increased tissue adherence, cellular emigration through draining lymphatics, or cell death.^53^ Here we found that the two subsets of corneal macrophages undergo opposite dynamic changes during the corneal healing process: ample CCR2^+^ corneal macrophages exist in the cornea 12 h after epithelial abrasion, which is also the time at which a large number of infiltrating neutrophils are present^54^ and CCR2^−^ corneal macrophages are undetectable. Thus, CCR2^+^ corneal macrophages are similar to inflammatory monocyte-derived macrophages in terms of their response at the early stage of peritoneal inflammation, and CCR2^−^ corneal macrophages resemble the F4/80^high^ peritoneal-resident macrophages, which undergo the *disappearance reaction* during the early stage of peritoneal inflammation. This result indicates that CCR2^+^ and CCR2^−^ corneal macrophages may have different roles in corneal wound healing.

Macrophages can be categorized as being of the classical, M1, activation state or the alternative, M2, activation state. M1 macrophages can secrete proinflammatory cytokines and promote the process of inflammation, whereas M2 macrophages can inhibit inflammation and are involved in the repair and remodeling of damaged tissues.^21, 22, 23, 24^ Quantitative PCR (qPCR) analysis of flow cytometry-sorted corneal macrophages showed that CCR2^+^ corneal macrophages express representative genes of M1 macrophages, and CCR2^−^ corneal macrophages express representative genes of M2 macrophages. Moreover, after corneal epithelial injury, depletion of CCR2^+^ corneal macrophages caused decreased recruitment of neutrophils and expression of inflammatory cytokines, whereas depletion of CCR2^−^ corneal macrophages resulted in increased neutrophil recruitment and expression of inflammation cytokines compared with the undepleted control group. As expected, both treatments delayed corneal wound healing. Therefore, these results indicate that CCR2^+^ corneal macrophages promote inflammation at the early stage of corneal wound healing and CCR2^−^ corneal macrophages inhibit inflammation during the later stage. Both macrophage populations are important for the healing of damaged corneal epithelium, and deficiency in either one result in an imbalance in inflammation. However, in our CCR2^−^ macrophage depletion study, it should be noted that some dendritic cells also express CSF1R, and thus injection of anti-CSF1R antibody may not just affect macrophages. Therefore, further study will be required to determine if dendritic cells also have a role in healing of the corneal epithelium.

Taken together, this study shows that corneal macrophages are a heterogeneous cell population, which can be classified into CCR2^−^ and CCR2^+^ populations. These populations migrate into cornea at different embryonic time points and have distinct phenotypes, gene expression profiles, and maintenance mechanisms. Moreover, the two populations balance the inflammatory responses during the corneal healing process to support the restoration of corneal integrity, and a deficiency in either one will delay the repair process. These findings have important significance for understanding the nature of corneal macrophages and their specific roles in corneal wound healing and provide new, potential therapeutic targets for the clinical treatment of defective wound healing in corneas such as is caused by diabetes and chronic microbial infection of the cornea.

## Methods

### Animals

SPF C57BL/6 mice that were free of eye disease were purchased from the Medical Experimental Animal Center (Guangdong, China). Rosa-YFP male mice were purchased from the Shanghai South Model Biological Technology Limited Company. These animals were raised at the Jinan University Animal Center. Experiments were performed using mice aged E12.5–17.5 or postnatal 7–8 weeks. One cornea was harvested from each mouse. All animal protocols were approved by the Jinan University Laboratory Animal Committee on Animal Welfare. All the animal treatments were in accordance with the Association for Research in Vision and Ophthalmology’s Statement for the Use of Animals in Ophthalmology and Vision Research as well as the guidelines of the Animal Experimental Committee at Jinan University. The animals were anesthetized using inhalation of 2% isoflurane and killed by overdose of CO_2_ and cervical dislocation.

### Corneal wound healing model

The wound-healing model of the cornea was as described previously.^54, 55, 56, 57, 58, 59^ After mice were anesthetized, the central zone of the corneas was marked by a trephine with a 2 mm diameter under the dissecting microscope. Then, the marked corneal epithelium was scraped with a golf club-like scraper. The wound area was stained with sodium fluorescein, and the healing process was analyzed by observing the change in the stained area.

### Bone marrow transplantation model

Recipient female mice drank gentamicin water (100 mg l^−1^) for 1 week before transplantation. Then, their hematopoietic systems were destroyed by a 5-Gy dose of radiation. To avoid eye tissues of mice being affected by radiation, each eye was covered with a lead cover. After radiation, bone marrow cells from the Rosa-YFP male mice were given to the recipient female mice through tail vein injection within 4 h. The recipient female mice then drank gentamicin water continuously. At 2 weeks after transplantation, the *Sry* gene, located on the Y chromosome, and the percentage of YFP^+^ cells were analyzed in the peripheral blood leukocytes of the recipient female mice to assess whether the bone marrow transplantation was successful.

### Macrophage depletion

To deplete CCR2^−^ corneal macrophages, 5 μl of rat anti-mouse CSF1R antibody (eBioscience, San Diego, CA; no. 14-1152-85) (0.1 μg μl^−1^), after being dialyzed in phosphate-buffered saline (PBS), was injected into each eye subconjunctivally (10 times, once every other day). The control group was injected with isotype rat immunoglobulin G2a (eBioscience; no. 14-4321-85). To deplete the CCR2^+^ corneal macrophages, 1 mg of the CCR2 antagonist, BMS CCR2 22 (R&D Systems, Minneapolis, MN; no. 3129), was dissolved in 1 ml of absolute ethyl alcohol to prepare the stock solution. The stock solution was adjusted with saline to 0.1 μg μl^−1^, and then each mouse was intraperitoneally injected with 10 μg per 20 g body weight (10 times, once every other day). The control group was injected with the diluent of BMS CCR2 22.

### Immunostaining

Immunostaining was carried out on the whole-mount corneas as described previously.^54, 55, 56, 57, 58, 59^ In brief, after the mice were killed, the eyeballs were detached and fixed in 4% paraformaldehyde for 1 h. The fixed eyeballs were placed in PBS and clipped under a dissecting microscope, leaving the whole corneas and limbi. The corneas were blocked with 2% bovine serum albumin for 15 min and then permeabilized with 0.1% Triton X-100/2% bovine serum albumin for 15 min. The treated corneas were then incubated with a mixture of antibodies (dilution 1:100), including anti-mouse CD64 conjugated with phycoerythrin (PE) (BioLegend, San Diego, CA; no. 139304), anti-mouse CCR2 conjugated with allophycocyanin (R&D Systems; no. FAB5538A), and anti-mouse F4/80 conjugated with fluorescein isothiocyanate (FITC) (eBioscience; no. 11-4801-82) or anti-mouse/rat Ki-67 conjugated with FITC (eBioscience; no. #11-5698-82) at 4 °C overnight. After being washed three times in PBS (5 min each time), the corneas were placed on glass slides and radially cut to flatten them. A fluorescent mounting medium containing 1 μM DAPI (4′, 6-diamidino-2-phenylindole) (Sigma-Aldrich, St. Louis, MO; no. 28718-90-3) was placed on the corneas. Image analysis of the corneas was performed with the DeltaVision Elite Microscopy Imaging System (Applied Precision, Issaquah, WA).

### Flow cytometric analysis

Flow cytometric analysis was performed as described previously.^60^ To avoid contamination by blood cells, PBS was flushed through the left ventricle of adult C57BL/6 mice. The eyeballs were detached and clipped under a dissecting microscope, leaving the whole corneas and limbi. The corneal tissues were cut into pieces and digested with 0.2% collagenase type I (Sigma-Aldrich; no. C0130) for 1.5–2 h. The digested cells were washed two times with PBS and passed through a 75 μm filter to obtain single cells. These single cells were blocked in Flow Cytometry Staining Buffer (eBioscience; no. 00-4222) containing anti-mouse CD16/32 antibody (eBioscience; no. 14-0161-85) at room temperature for 10 min, followed by incubation at room temperature for 30 min with a mixture of the following antibodies (dilution 1:100): anti-mouse CD45 antibody conjugated with FITC (BD Biosciences, San Jose, CA; no. 553080), anti-mouse CD64 conjugated with Brilliant violet 421 (BioLegend; no. 139309) (anti-mouse CCR2 (R&D Systems; no. FAB5538A) or CX3CR1 (BioLegend; no. 149007) or CD206 (BioLegend; no. 141707) conjugated with allophycocyanin), anti-mouse CD11b conjugated with Percp Cy5.5 (eBioscience; no. 45-0112-82) (anti-mouse F4/80 (eBioscience; no. 12-4801-82) or CD301 (R&D Systems; no. FAB4297P) conjugated with PE), anti-mouse Ly6C conjugated with PE-Cy7 (eBioscience; no. 25-5932-82), and anti-mouse MHC-II conjugated with Alexa Fluor 700 (eBioscience; no. 56-5321-82).

To detect the intracellular antigen Ki-67, cell suspensions, stained with the indicated antibodies against the cell surface antigen, were fixed in 4% paraformaldehyde for 30 min and permeabilized with 0.1% Triton X-100 for 10 min. After being washed two times with PBS, the cell suspensions were incubated with anti-mouse/rat Ki-67 antibody conjugated with PE (eBioscience; no. 12-5698-82) (dilution 1:100) at room temperature for 30 min. Before the samples were analyzed with the BD FACSVerse, we used the Anti-Rat and Anti-Hamster Igκ/Negative Control Compensation Particles Set (BD Biosciences; no.552845) to adjust the fluorescence compensation.

### Transcript amplification in corneal macrophages

Corneal cell suspensions, digested with 0.2% collagenase type I (Sigma-Aldrich; no. C0130), were stained with a mixture of antibodies (dilution 1:100) including anti-mouse CD45 antibody conjugated with FITC (BD Biosciences; no. 553080), anti-mouse CD64 conjugated with Brilliant violet 421 (BioLegend; no. 139309), and anti-mouse CCR2 conjugated with allophycocyanin (R&D Systems; no. FAB5538A) at room temperature for 30 min. These stained corneal cells were sorted by flow cytometry using the BD FACSAria to obtain CD45^+^ CD64^+^ CCR2^−^ and CD45^+^ CD64^+^ CCR2^+^ macrophages. Then, the whole transcriptomes of the CCR2^−^ and CCR2^+^ corneal macrophages were amplified using the REPLI-gWTA Single Cell Kit (Qiagen; no. 150063).

### qPCR

The corneal tissues were cut into pieces, placed in Buffer RZ (Tiangen, Beijing, China; no. RK145), and smashed using a TissueRuptor (Qiagen, Germantown, MD). The total RNA of the corneal tissues was obtained with the RNA simple Total RNA Kit (Tiangen; no. DP419). Then, cDNA was generated using the ReverTra Ace qPCR RT Kit (Toyobo, Osaka, Japan; no. FSQ-101). Finally, the relative expression of the target genes in the corneal cDNA and the amplified transcriptomes of the sorted CCR2^−^ and CCR2^+^ corneal macrophages were detected using the THUNDERBIRD SYBR qPCR Mix (Toyobo; no. QPS-201). The PCR primers used in this study are shown in Table 1.

### Statistical analyses

The results are presented as the mean±s.d. For comparisons between groups, factorial design analysis of variance and unpaired Student’s *t*-test were performed. Statistical significance was set at *P*<0.05.

## Figures and Tables

**Table 1:** PCR primers used in this study

**Gene name**	**Primer pair (5′–3′)**	
*Irf7*	Forward	CACAGTCTTCCGCGTACCCT
	Reverse	GTCTTCCAGCCTCTTCGCTCT
		
*Nr1h3*	Forward	ATTCTTCCGCCGCAGTGTCA
	Reverse	TGTTCCTCTTCTTGCCGCTTC
		
*Hdac10*	Forward	CTCCCACTGGCCTTCGAGT
	Reverse	CCCTCCAACACAGCACAAATCCG
		
*Taf7*	Forward	ATGAATCCGACGAGCAACACC
	Reverse	AATGAGATCTTCCTGGCGCTT
		
*Gata2*	Forward	CCAGACCCCAGCACAACAGGA
	Reverse	GCCGCCTTCCATCTTCATGCTCT
		
*Csf1r*	Forward	AGATCTTCTCGCTTGGTCT
	Reverse	TGTATATGTTCTTCGGTGCAA
		
*IL-1β*	Forward	TTTGAAGTTGACGGACCCCAA
	Reverse	TCATATGGGTCCGACAGCAC
		
*TNF*	Forward	AAAATTCGAGTGACAAGCCT
	Reverse	CTTTGAGATCCATGCCGTTG
		
*IL-10*	Forward	ACAACATACTGCTAACCGACT
	Reverse	AGAAATCGATGACAGCGCCTC
		
*Arg1*	Forward	ATCAACACTCCCCTGACAACC
	Reverse	CCATCACCTTGCCAATCCC
		
*Mrc1*	Forward	CGTGGATTCCTTTCTATGGC
	Reverse	ACACAATCATTCCGTTCACCA
		
*Mgl2*	Forward	AAGAGCCATTTTAGACAACACC
	Reverse	AGTTCCTGCCTGTGATCCTC
		
*Sry*	Forward	AGGTGGAAAAGCCTTACAGAA
	Reverse	GGATATCAACAGGCTGCCAAT
		
*GAPDH*	Forward	CAAGGACACTGAGCAAGAG
	Reverse	TGCAGCGAACTTTATTGATG

## Supplementary Material

Supplementary Information

Supplementary Figure1

Supplementary Figure2
