# Supplementary material for: CCR2− and CCR2+ corneal macrophages exhibit distinct characteristics and balance inflammatory responses after epithelial abrasion
Source: Mucosal Immunol. 2017 Jan 25;10(5):1145–59. doi: 10.1038/mi.2016.139 (PMC5562841; doi:10.1038/mi.2016.139)
Supplement: Supplementary Information [file mi2016139x1.doc]

**CCR2**^−^ **and CCR2^+^ corneal macrophages exhibit distinct characteristics and balance inflammatory responses after epithelial abrasion**

Jun Liu^1,2,3^, Yunxia Xue^2^, Dong Dong^2^, Chengju Xiao^3^, Cuipei Lin^2^, Hanqing Wang^3^, Fang Song^3^, Ting Fu^2^, Zhaorui Wang^4^, Jiansu Chen^2^, Hongwei Pan^2^, Yangqiu Li^3^, Dongqing Cai^3^, Zhijie Li^2,3,5*^

^1^Integrated Chinese and Western Medicine Postdoctoral Research Station, Jinan University, Guangzhou, China; ^2^International Ocular Surface Research Center and Institute of Ophthalmology, Jinan University Medical School, Guangzhou, China; ^3^Key Laboratory for Regenerative Medicine, Ministry of Education, Jinan University, Guangzhou, China; ^4^Department of Medical Images, The Third People’s Hospital, Puyang, China; ^5^Section of Leukocyte Biology, Department of Pediatrics, Children’s Nutrition Research Center, Baylor College of Medicine, Houston, Texas.

^*^Corresponding author; email address: zhijiel@bcm.edu or zhijielee@yahoo.com

**Supplementary information**


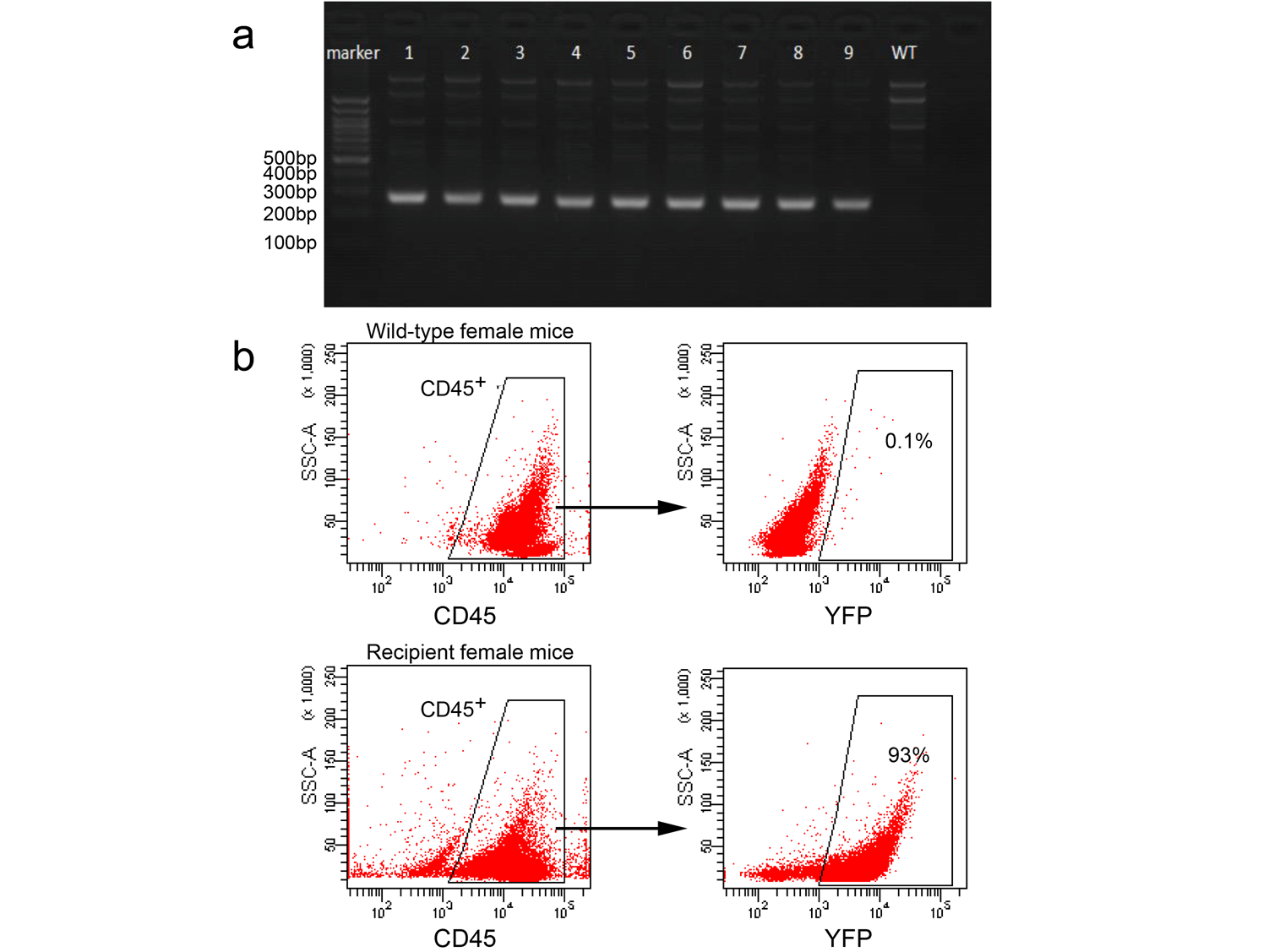


**Supplementary Figure 1. Verification of bone marrow transplantation.** Bone marrow from male Rosa-YFP mice was transplanted into irradiated wild-type C57BL/6 female mice. (a) PCR analysis of Sry gene (1～8: recipient female mice; 9: male mouse; WT: wild-type female mouse). (b) Quantitation of YFP^+^ cells. Percentage of YFP^+^ cells in peripheral blood leukocytes from wild-type and recipient female mice was measured using flow cytometry.


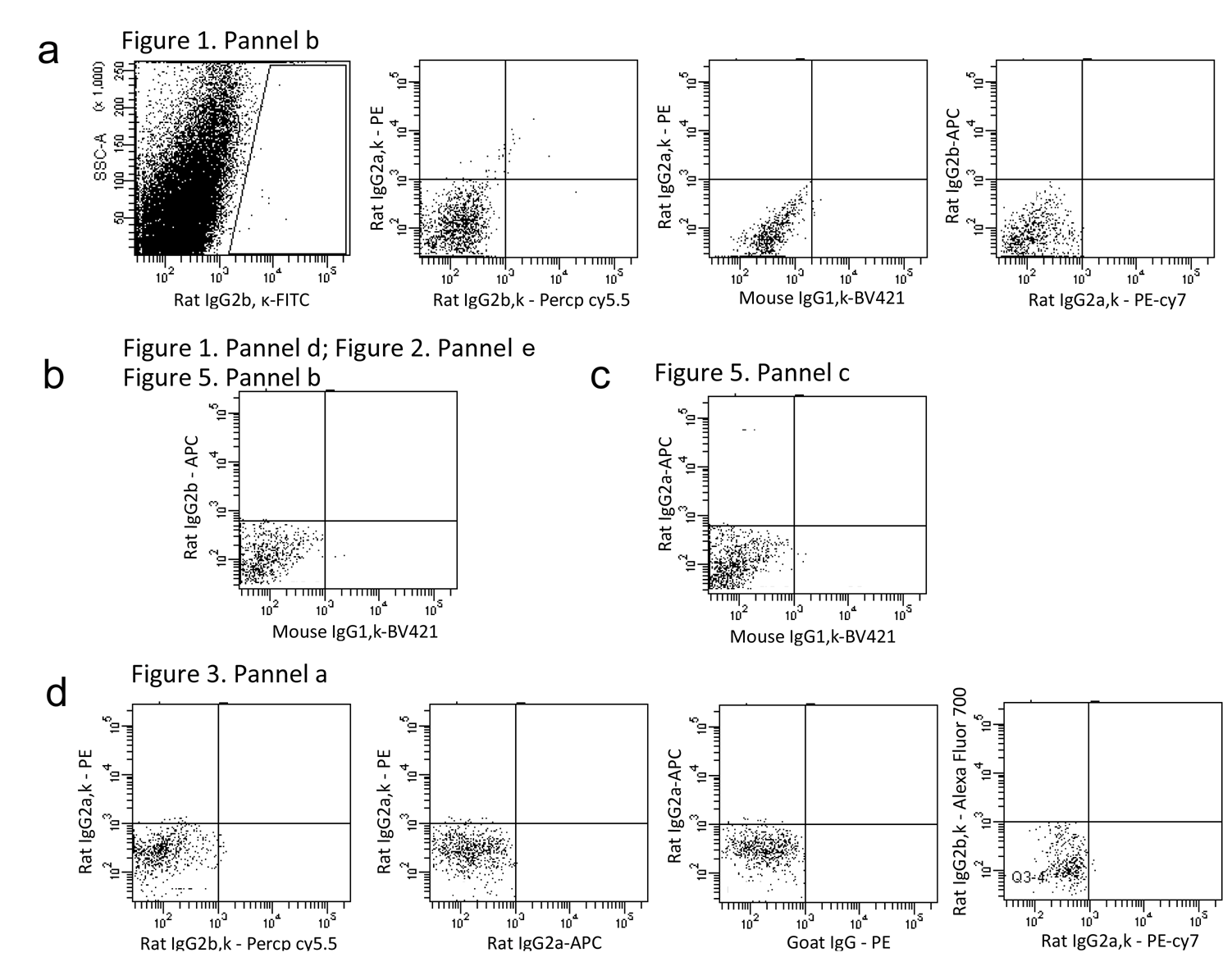


**Supplementary Figure 2. Isotype controls for justifying the gating strategies.** (a) Isotype controls for antibodies (CD45-FITC, F4/80-PE, CD11b-Percp cy5.5, CD64-BV421, CCR2-APC and Ly6C-PE-cy7) used in the panel b of figure 1. (b) Isotype controls for antibodies (CCR2-APC and CD64-BV421) used in the panel d of figure1, panel e of figure 2, and panel b of figure 5. (c) Isotype controls for antibodies (CD206-APC and CD64-BV421) used in the panel c of figure 5. (d) Isotype controls for antibodies (F4/80-PE, CD11b-Percp cy5.5, CX3CR1-APC, CD206-APC, CD301-PE, MHC-II-Alexa Fluor 700 and Ly6C-PE-cy7) used in the panel a of figure 3.
