# Supplementary figures and images for: CCR2− and CCR2+ corneal macrophages exhibit distinct characteristics and balance inflammatory responses after epithelial abrasion
Source: Mucosal Immunol. 2017 Jan 25;10(5):1145–59. doi: 10.1038/mi.2016.139 (PMC5562841; doi:10.1038/mi.2016.139)

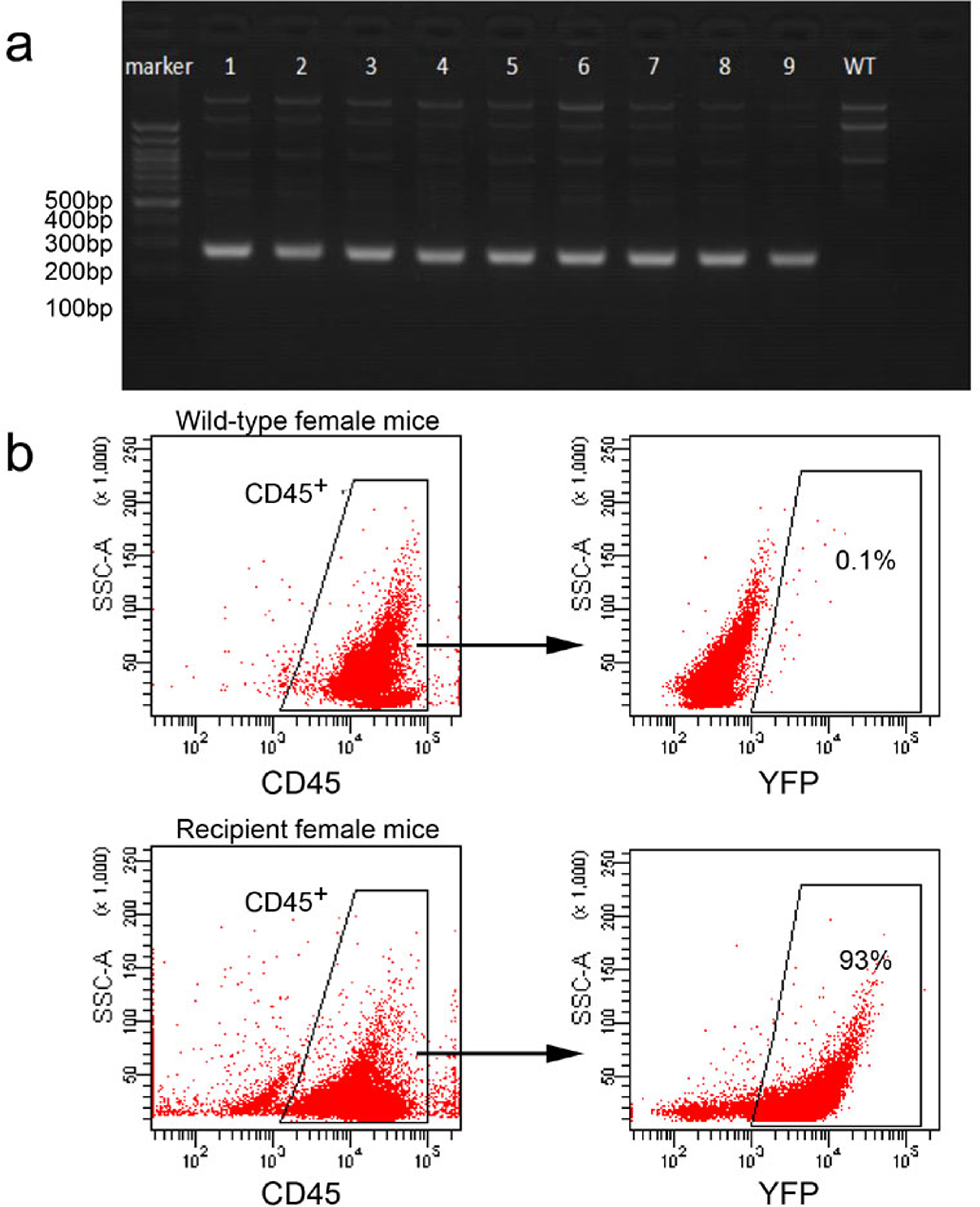

Supplement: Supplementary Figure1 [file mi2016139x2.tif]

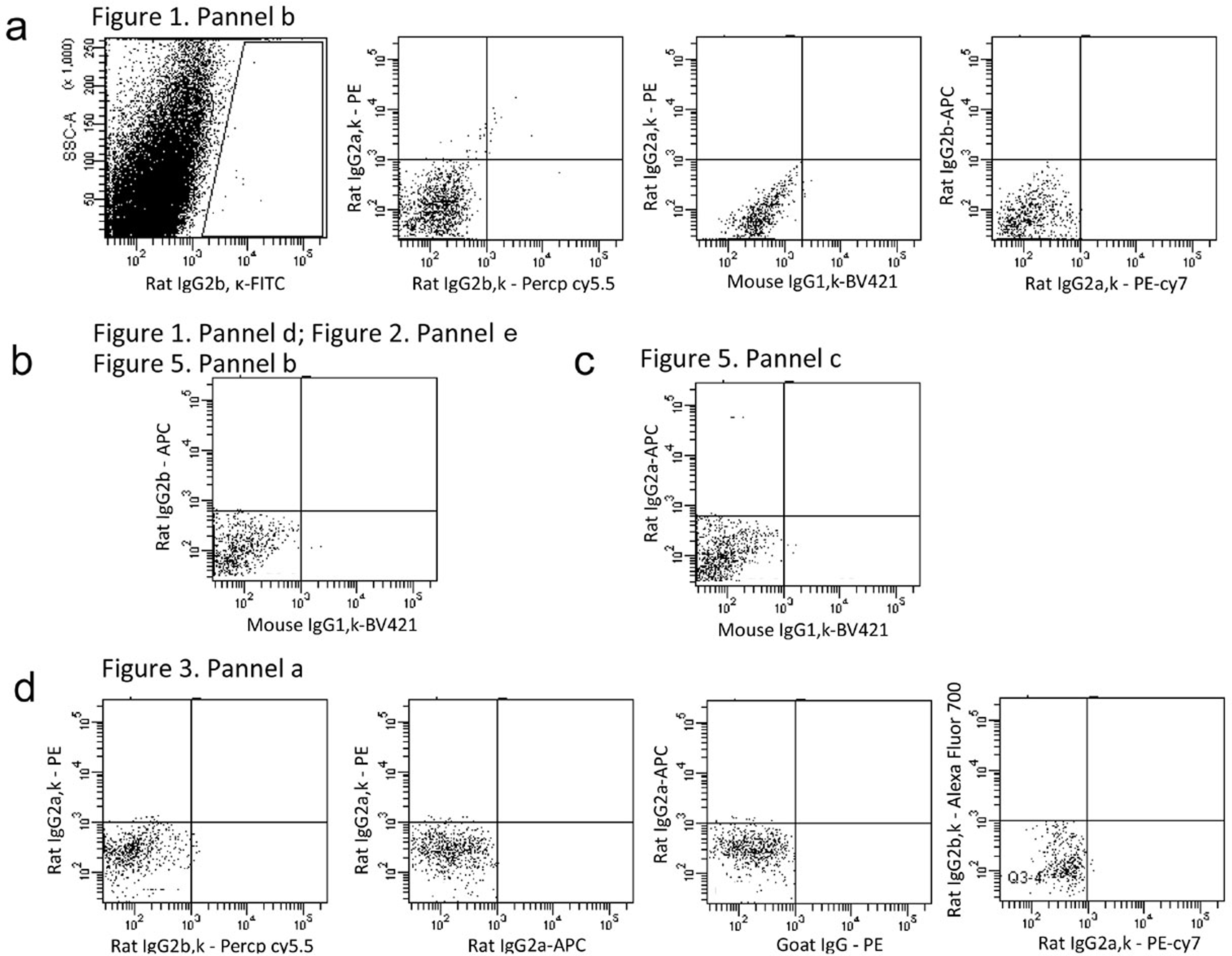

Supplement: Supplementary Figure2 [file mi2016139x3.tif]
